# Supplementary material for: Size-dependent strong metal-support interaction in TiO2 supported Au nanocatalysts
Source: Nat Commun. 2020 Nov 16;11:5811. doi: 10.1038/s41467-020-19484-4 (PMC7669859; doi:10.1038/s41467-020-19484-4)
Supplement: Supplementary file 1 — Supplementary Information [file 41467_2020_19484_MOESM1_ESM.pdf]

Supplementary Information

# Size-dependent strong metal-support interaction in $TiO_2$ supported Au nanocatalysts

Xiaorui Du et al.

## 1. Supplementary Figures and Tables

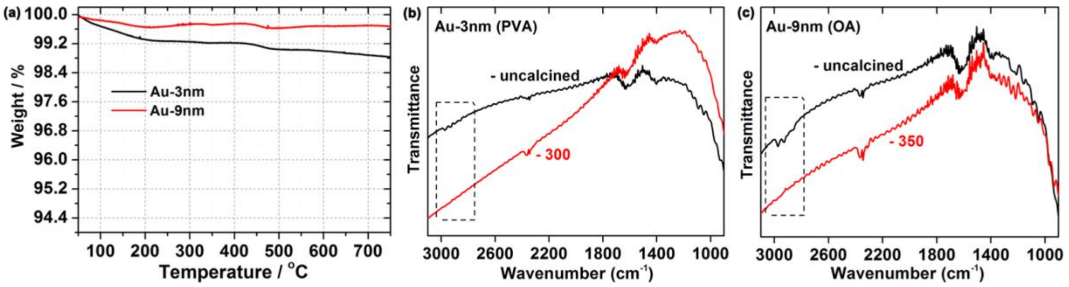


**Supplementary Figure 1**. (a) Thermogravimetric (TG) analysis of the Au-3nm and Au-9nm samples that have been calcined at 300 and 350 $^oC$ under air. During the TG analysis, samples were heated to 800 $^oC$ in air flow (100 mL $min^{-1}$) with a ramp rate of 10 $^oC$/min. (b, c) FT-IR spectra of (b) PVA and (c) OA protected Au/$TiO_2$ and corresponding calcined sample.

As shown in **Supplementary Figure 1a**, the weight loss is negligible (< 0.4%) after 300 $^oC$ for both samples, indicating the inexistence of residual ligands; the slight weight loss below 200 $^oC$ should be related to desorption of the surface adsorbed water. In addition, the FT-IR spectra (**Supplementary Figure 1b** and **c)** show that the bands associated with the C-H stretching vibrations (2850 - 2980 $cm^{-1}$) in OA[1,2] and PVA[3,4] disappeared after calcination, reinforcing the successful removal of the ligands from the supported Au NPs.

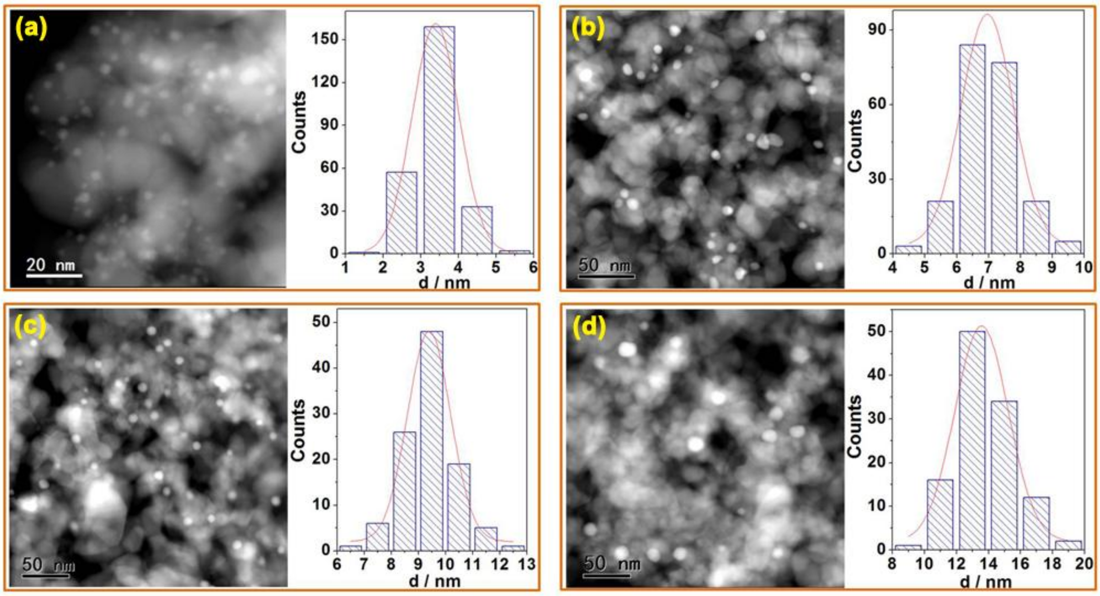


**Supplementary Figure 2.** The high-angle annular dark-field scanning transmission electron microscopy (HAADF-STEM) images and corresponding Au particle size distributions of the as-synthesized samples; (a) Au-3nm, (b) Au-7nm, (c) Au-9nm, and (d) Au-13nm. The size distributions were determined by analyzing 150-300 Au NPs from corresponding HAADF-STEM and TEM images, and the mean diameters of Au NPs within the samples were 3.4 ± 1.0, 7.0 ± 1.2, 9.4 ± 1.6, and 13.6 ± 2.0 nm, respectively.

**Supplementary Table 1.** A summary of synthesis information and SMSI performance for samples.

| Samples | protecting agent | calcination temperature (°C) | Au loadings (wt%) [a] | particle size distribution [b] | completely encapsulated reduction temperature (°C) |
|---|---|---|---|---|---|
| Au-3nm | PVA | 300 | 0.59% | 3.4±1 nm | 600 |
| Au-7nm | PVA | 450 | 1.20% | 7±1.2 nm | 500 |
| Au-9nm | Oilamine | 350 | 1.20% | 9.4±1.6 nm | 400 |
| Au-13nm | Oilamine | 400 | 1.50% | 13.6±2 nm | 400 |
| Au-3+9nm | PVA & Oilamine | 350 | 1.46% [c] | Shown in **Supplementary Figure 14** | unmeasured |

[a] Determined by inductively coupled plasma optical emission spectrometer (ICP-OES).

[b] The size distributions were determined by analyzing 150-300 Au NPs from corresponding HAADF-STEM and TEM images.

[c] The loading amount of ~3 nm Au NPs in this sample is 0.25%, determined by ICP-OES.

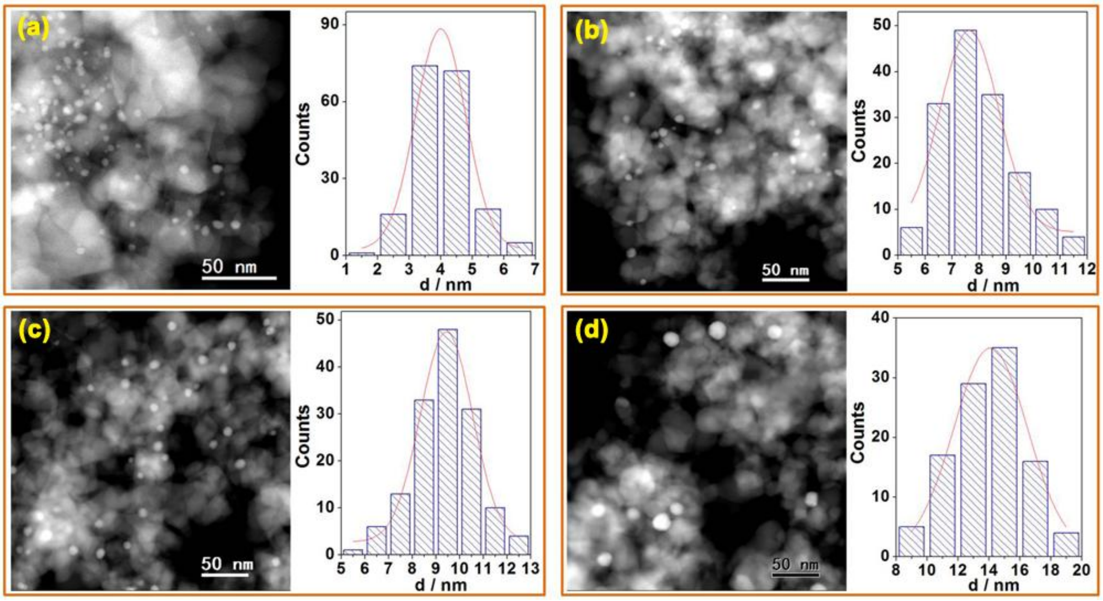


**Supplementary Figure 3.** The HAADF-STEM images and corresponding Au particle size distributions of each sample after high-temperature reduction; (a) Au-3nm-H600, (b) Au-7nm-H500, (c) Au-9nm-H400, and (d) Au-13nm-H400. The size distributions were determined by analyzing 150-300 Au NPs from corresponding HAADF-STEM and TEM images.

Note: The mean diameters of Au NPs within the reduced samples were 4.0 $\pm$ 1.0, 7.6 $\pm$ 1.4, 9.4 $\pm$ 1.6, and 14.1 $\pm$ 2 nm, respectively.

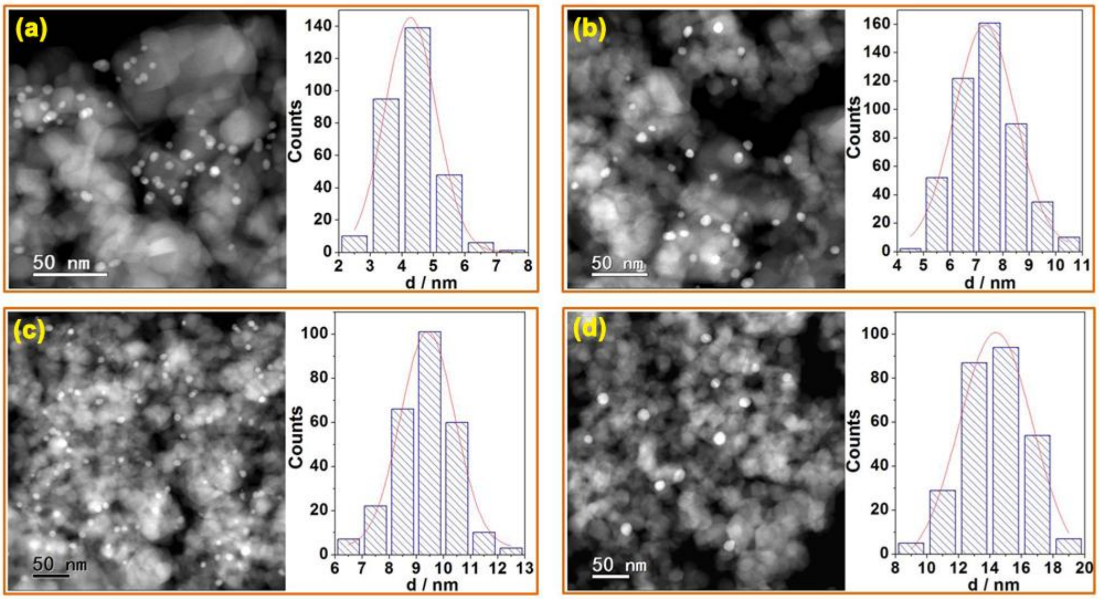


**Supplementary Figure 4.** The HAADF-STEM images and corresponding Au particle size distributions of each sample after re-oxidation at 400 °C; (a) Au-3nm-H600-O400, (b) Au-7nm-H500-O400, (c) Au-9nm-H500-O400, and (d) Au-13nm-H500-O400. The size distributions were determined by analyzing 200-300 Au NPs from corresponding HAADF-STEM and TEM images.

Note: The mean diameters of Au NPs within the re-oxidation samples were 4.3 ± 1.0, 7.3 ± 1.5 , 9.6 ± 1.8, and 14.4 ± 2 nm, respectively.

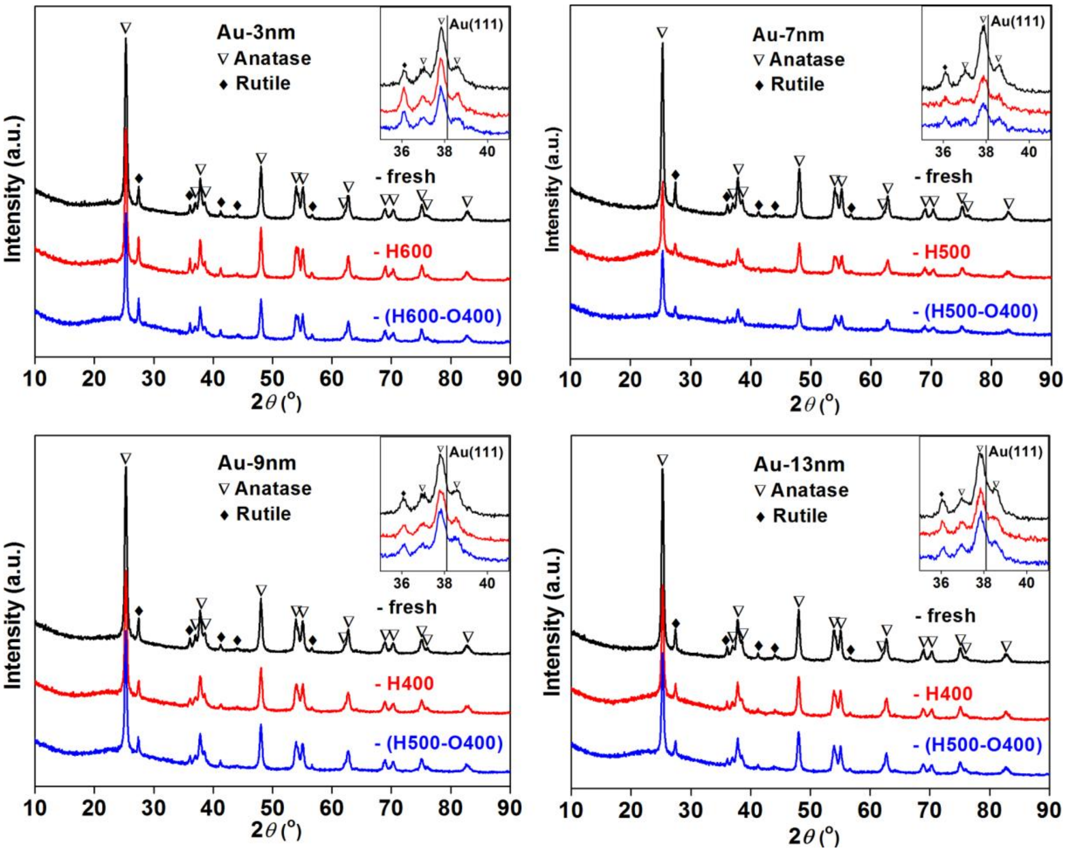


**Supplementary Figure 5.** XRD patterns of each sample before and after high-temperature reduction or re-oxidation. It can be seen that the heat treatment did not impact the crystal phase composition of $TiO_2$, which is in consistent with the previous report. Inset of each pattern is the enlarged partial XRD patterns at the range of 35~41 °.

The marked Anatase phase was referenced from PDF#21-1272, and Rutile phase was referenced from PDF#21-1276.

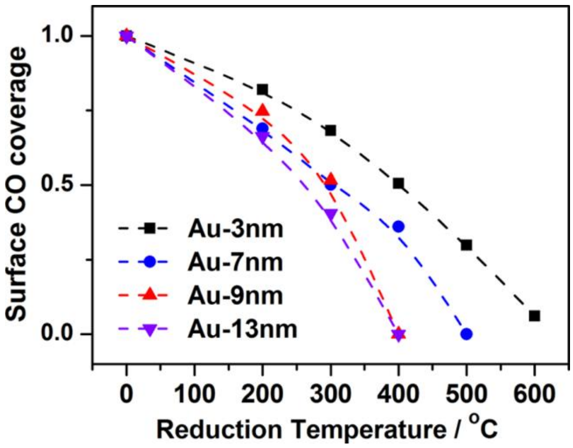


**Supplementary Figure 6**. Normalized size-dependent CO coverage following reduction at different temperatures based on results in **Figure 1**.

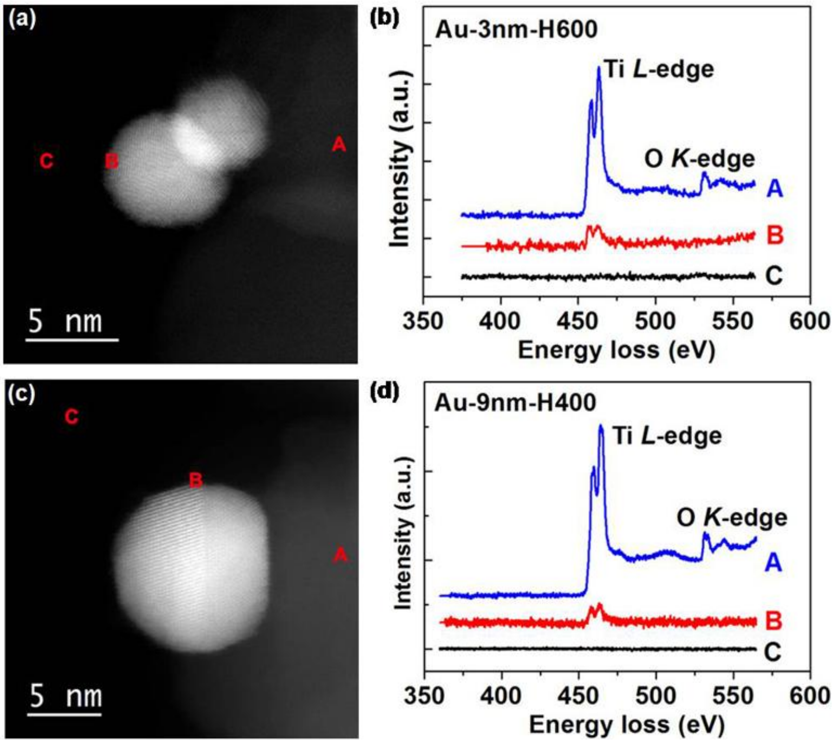


**Supplementary Figure 7.** EELS spectra of (a-b) Au-3nm-H600 and (c-d) Au-9nm-H400 sample. (a, c) the high resolution HAADF-STEM images of (a) Au-3nm-H600 and (c) Au-9nm-H400; (b, d) the corresponding EELS spectra of (b) Au-3nm-H600 and (d) Au-9nm-H400. The spectra were background-subtracted.

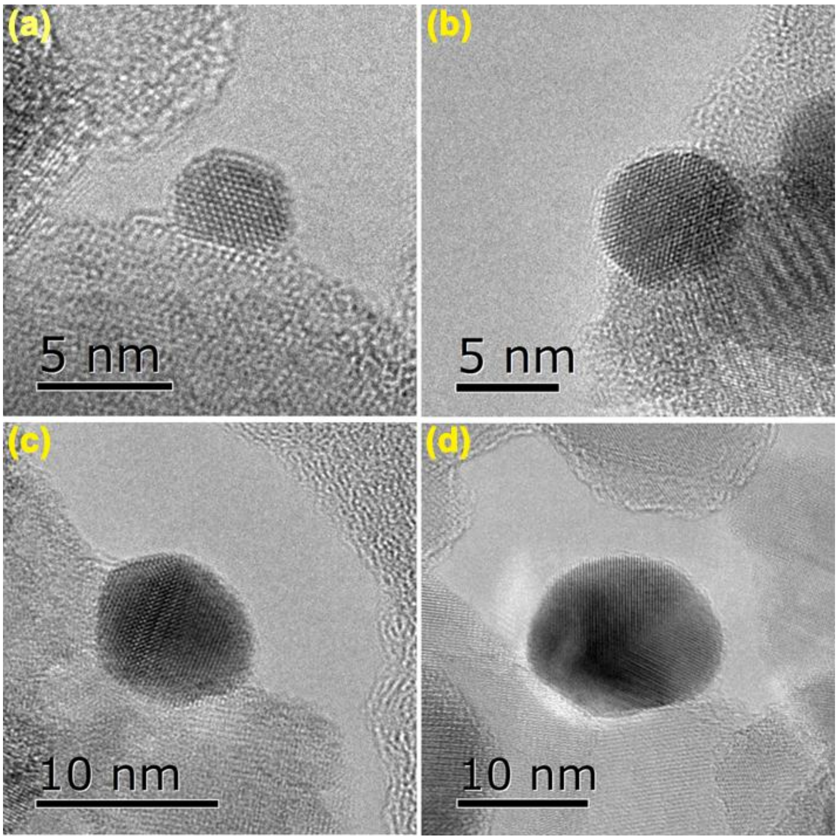


**Supplementary Figure 8.** The representative high-resolution transmission electron microscopy (HRTEM) images of the samples re-oxidized at 400 $^{o}$C; (a) Au-3nm-H600-O400, (b) Au-7nm-H500-O400, (c) Au-9nm-H500-O400, and (d) Au-13nm-H500-O400.

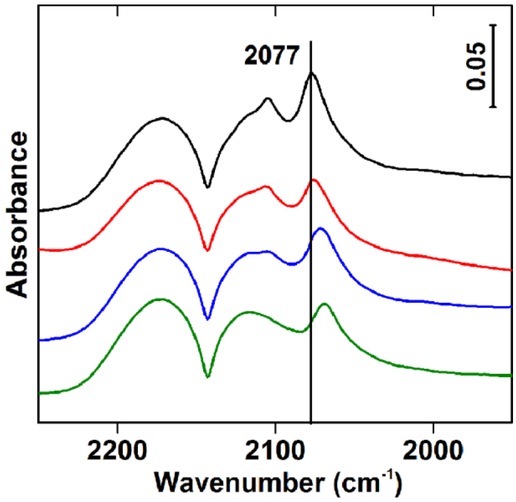


**Supplementary Figure 9.** *In situ* DRIFT spectra of CO adsorption on (a) Au-3nm-H300, (b) Au-7nm-H300, (c) Au-9nm-H300, and (d) Au-13nm-H300 samples. Their CO-$Au^{x-}$ bands are at 2,077, 2,075, 2,071, and 2,068 $cm^{-1}$, respectively.

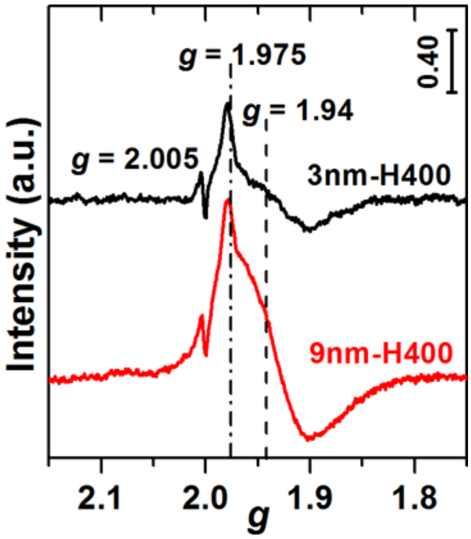


**Supplementary Figure 10**. EPR spectra of the Au-3nm-H400 and Au-9nm-H400 samples obtained at 110 K.

The signals with $g$ = 2.005 were ascribed to the typical of an $O^-$ ion (oxygen-centered surface hole trapping sites),[5-7] whose intensity remained unchanged to the samples. The signals with $g$ = 1.975 were attributed to bulk $Ti^{3+}$ defects.[8,9] And the signals centered at $g$ = 1.94, with a broadening characteristic, were ascribed to surface $Ti^{3+}$ after high-temperature reduction.[9-11]

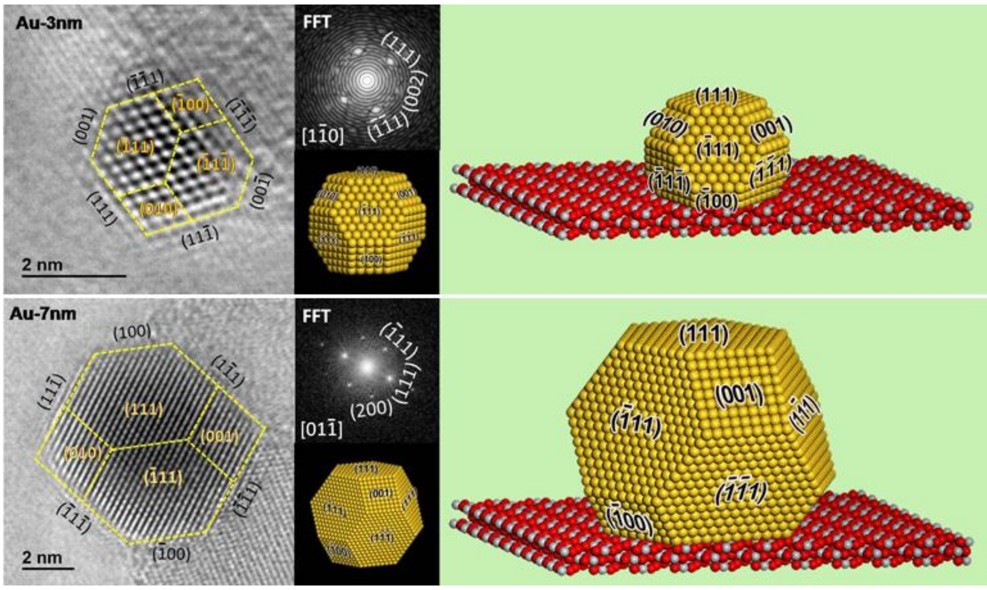


**Supplementary Figure 11**. Representative HRTEM images and corresponding geometric structure analysis for the samples.

It can be observed that even calcined at different temperature, the Au NPs had similar shape of truncated octahedron for Au-3nm and Au-7nm, with Au {111} and {100} facets mainly exposed, consisting with the equilibrium shape of *fcc* metals.[12,13]

## 2. Supplementary information for the discussion of surface energy

**Supplementary Table 2.** Comparison of surface energies of different metals and $TiO_2$. $\gamma$ in J $m^{-2}$.

| | $\gamma_{sv0}$ [a] | $\gamma_{sv0}$(E) [b] |
|---|---|---|
| Au (111) | 1.52 | 1.51, 1.50 |
| Ag (111) | 1.20 | 1.25, 1.25 |
| Cu (111) | 1.83 | 1.79, 1.83 |
| Pd (111) | 1.85 | 2.00, 2.05 |
| Ni (111) | 2.44 | 2.38, 2.45 |
| Pt (111) | 2.54 | 2.49, 2.48 |
| Rh (111) | 2.70 | 2.66, 2.70 |
| Ir (111) | 3.19 | 3.05, 3.00 |
| Ti (0001) | 1.96 | 1.99, 2.10 |
| $TiO_2$ [c] | 1.93, 1.66, and 1.34 [d] | 0.5-1.7 [e], or < 0.7[f] |

[a] The predicted values of $\gamma_{sv0}$ by Jiang and Lu *et. al* as in literatures.[14-16]

[b] The experimental results of $\gamma_{sv0}$ value from literatures.[17,18]

[c] It is reported that the surface energy of $TiO_2$ are different depending on its crystal form.[19]

[d] The listed values are for rutile, brookite, and anatase, respectively, reported by literature (20).[20]

[e] Reported by literature (21).[21]

[f] Reported by literature (22).[22]

The intrinsic/bulk surface energy (also the solid-vapor interface energy, $\gamma_{sv0}$, a intrinsic physical quantity) of different metals are listed in **Supplementary Table 2**. The $\gamma_{sv0}$ value of Au is obvious lower than that of Ir, Rh, Pt, Ni, and Pd. However, the reported surface energy value of $TiO_2$, either calculated or experimental, are not uniform to be used as one parameter to compare with that of Au.

In nanoscale, size-dependence of surface tension is significant and has been well studied since Tolman[23]. Based on the long-term study of several research groups,[14,15,24-26] the size-dependent solid-vapor interface energy $\gamma_{sv}(d)$ could be approximately theoretical determined by **Supplementary Equation (1)**, where $d$ represents the diameter of Au NPs, and C represents a constant varied with different modeling method. It can be seen that the $\gamma_{sv}(d)$ value will increase with increasing of particle size if C is positive. In our system, this implies

a potential driving force for the size-dependent SMSI: the minimization of the surface free energy of Au NPs.

$$\gamma_{sv}(d) \propto \gamma_{sv,\infty}\ (1\text{-}C/d) \tag{1}$$

However, a few reports suggested an opposite trend mostly either simulation at 0K[27] or experimentally measured at low temperature[28]. A recent work by Molleman and Hiemstra performed a comprehensive study on the size-dependence of surface tension at nanoscale,[29] which is helpful to address the controversy. They showed that at high-temperatures positive relationship between particle size and surface tension is possible, as shown in **Supplementary Figure 12**. Since the temperature involved in our system is ≥ 400 °C, applying this *positive* relationship of size-dependent surface tension is reasonable. (For Au NP, see ref.[29]; For $TiO_2$, see ref.[30], estimation of interface surface tension $\gamma_{int,\infty}$ is based on criterion for fully spreading: $\gamma_{TiO2\text{-}x,\infty} + \gamma_{int,\infty} - \gamma_{Au,\infty} \leq 0$):

$$\gamma_{TiO_{2-x}} = \gamma_{TiO_{2-x},\infty}\left(1 - \frac{4\delta_{TiO_{2-x}}}{r}\right) \tag{2a}$$

$$\gamma_{int} = \gamma_{int,\infty}\left(1 - \frac{4\delta_{int}}{r}\right) \tag{2b}$$

$$\gamma_{Au} = \gamma_{Au,\infty}\left(1 - \frac{4\delta_{Au}}{r}\right) \tag{2c}$$

$$\gamma_{Au} - \gamma_{TiO_{2-x}} - \gamma_{int} = \Delta\gamma_{\infty} - 4\frac{\Delta(\delta\gamma_{\infty})}{r} \tag{2d}$$

For rough estimation, $\Delta\ (\delta\ \gamma_{\infty})$ = 1.3212 J/m is positive (see **Supplementary Table 2** and, Au-Au bond length is 2.88 Angstrom, Ti-O bond length is 1.94 Angstrom, Au-Ti bond length is roughly 3 Angstrom), it is easy to find that surface tension difference, which can be understood as driving force of encapsulation, increases with nanoparticle size, although driving force cannot be used directly to predict encapsulation of NP, for driving force is not equivalent with encapsulation degree.

Therefore, it is speculated that the size-dependent SMSI is related to the size-dependency of the surface tension of Au NP. To manifest concrete variation illustration for this, an in-depth discussion and interpretation was done by establishing thermodynamic dynamic equilibrium for the encapsulation of Au by $TiO_2$.

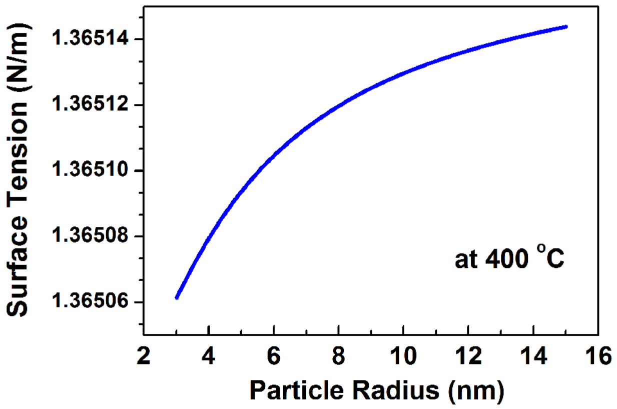


**Supplementary Figure 12**. Size-dependent surface tension calculated at 400 °C. Formulation, enthalpy, entropy and surface tension data used are from ref.[29].

## 3. Formulation and numerical solution of the Au-$TiO_2$ encapsulation model

Based on our observation of TEM image (**Figure 4a**), as shown in **Figure 4b**, we try to establish a model to qualitatively reproduce our experimental results. Two variables, *r* and $\theta$ are used to represent the state of NP. *r* is the radius of NP (a globular model particle is used for simplicity, different crystal shape will only cause a slight scaling of radius, see ref.[29]), $\theta$ represents the degree of encapsulation. At surfaces contacting point, surface tension balance Equation (1) is used:

$$\gamma_{Au} = \gamma_{int} + \gamma_{TiO_{2-x}}$$

Interface stress is defined as response of one surface excess Gibbs free energy, in **Supplementary Equation (3)**, where *A* denotes the area of interface[31].

$$f_{int} = \frac{\partial(\gamma_{int} A_{int})}{\partial A_{int}} = \gamma_{int} + A_{int}\frac{\partial \gamma_{int}}{\partial A_{int}} \qquad (3)$$

$$\frac{\partial \gamma_{int}}{\partial A_{int}} = \frac{\Delta \gamma_{int}}{\Delta A_{int}} \qquad (4)$$

for the part of Au surface that has been covered by $TiO_{2-x}$,

$$A_{int} = r^2 \int_0^{\theta} \sin\theta \, d\theta \int_0^{2\pi} d\phi \,, \;\; \Delta\varepsilon_{int} \equiv \Delta A_{int} / A_{int}$$

Then,

$$\gamma_{int}(r,\theta) = \frac{f_{int}\Delta\varepsilon_{int} + \gamma_{int,0}}{\Delta\varepsilon_{int} + 1} \qquad (5)$$

denoted that[32]

$$f_{int} = K_{int}(r,\theta)\gamma_{int,0}$$

$$\gamma_{int}(r,\theta) = \frac{K_{int}\Delta\varepsilon_{int} + 1}{\Delta\varepsilon_{int} + 1}\gamma_{int,0} \qquad (6)$$

Also for part of Au surface that is still bare,

$$\gamma_{Au}(r,\theta) = \frac{K_{Au}\Delta\varepsilon_{Au} + 1}{\Delta\varepsilon_{Au} + 1}\gamma_{Au,0} \qquad (7)$$

for simplicity, ($r$, $\theta$)-dependence of term $\gamma_{TiO2-x}$ is not considered, although for practical system, small $\gamma_{TiO2-x}$ is more favored for starting up mass transfer of overlayer species,

rearrangement after encapsulation is also reported[33], but in our system, we did not observe significant structural difference. Collecting equations above, relationship between $r$ and $\theta$ is obtained:

$$\frac{K_{Au}\left[2\left(1-\frac{r_0}{r}\right)-\frac{\Delta\theta\sin\theta}{1+\cos\theta}\right]+1}{2\left(1-\frac{r_0}{r}\right)-\frac{\Delta\theta\sin\theta}{1+\cos\theta}+1}\gamma_{Au,0}=\frac{K_{int}\left[2\left(1-\frac{r_0}{r}\right)+\frac{\Delta\theta\sin\theta}{1-\cos\theta}\right]+1}{2\left(1-\frac{r_0}{r}\right)+\frac{\Delta\theta\sin\theta}{1-\cos\theta}+1}\gamma_{int,0}+\gamma_{TiO_{2-x}} \quad (8)$$

**Supplementary Equation (8)** is solved numerically:

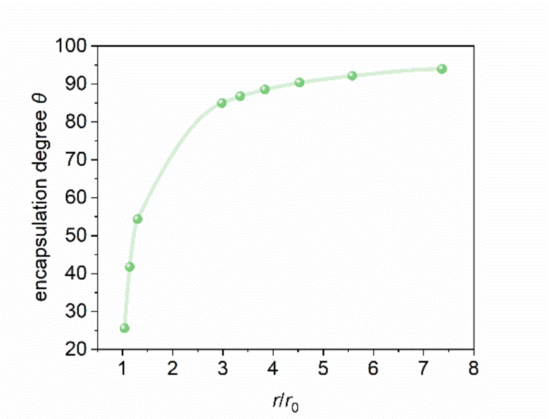


**Supplementary Figure 13**. A speculative curve plotted according to numerical solutions of **Supplementary Equation (8)**, where we set value $\gamma_{Au,0}$ = 1.50 J/m$^2$ (references **17** and **18**), $\gamma_{int,0}$ = 0.11 J/m$^2$, $\gamma_{TiO2-x}$ = 1.39 J/m$^2$ (anatase data is used), $K_{Au}$ = 5 (ref.[32]), $K_{int}$ = -1 (thin overlayer is treated as Au-Ti alloy[33,34], therefore negative value is used[32]). This model is failed to predict a fully-encapsulated state in finite scale of nanoparticle size, possible reasons are mainly: (1) neglect of nanoparticle sinking into support during encapsulation, (2) over-simplification of interface stress. However, both modification will bring tremendously high complexity into formulation, meanwhile, to obtain parameters for (1), large scale *ab initio* molecular dynamics simulation may be involved in future work.

## 4. Supplementary Figures and Tables for catalytic application

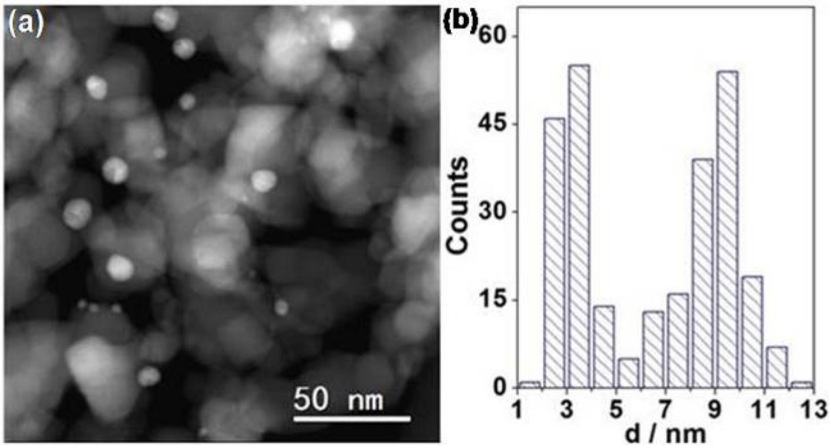


**Supplementary Figure 14.** The (a) HAADF-STEM image and (b) corresponding Au particle size distribution of Au-3+9 sample.

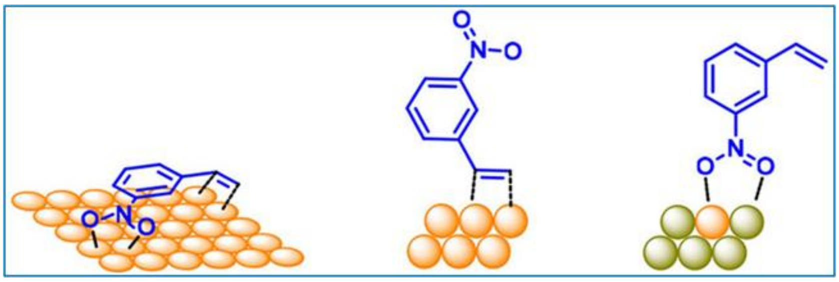

**Supplementary Figure 15**. Possible adsorption patterns of 3-nitrostyrene on catalysts with different geometric structures. Reproduced with permission from *Chem. Rev.* ***2020***, *120, 683-733*.[35] Copyright 2020 American Chemical Society.

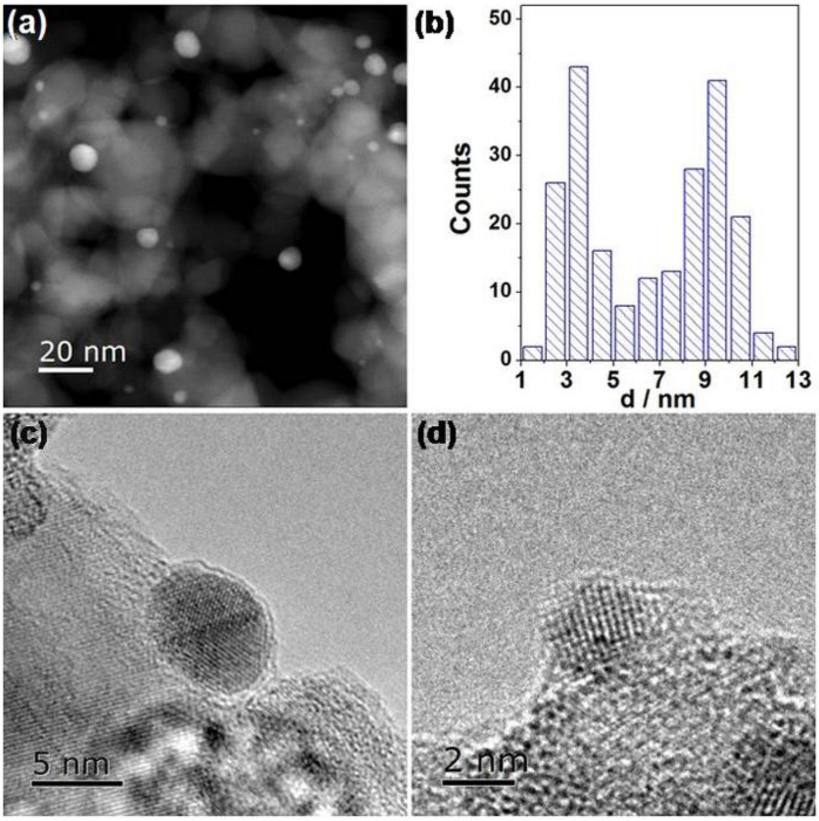


**Supplementary Figure 16**. The (a) HAADF-STEM image and (b) corresponding Au particle size distribution of Au-3+9nm-H400 sample; and the representative HRTEM image of the (c) large and (d) small particles in Au-3+9nm-H400.

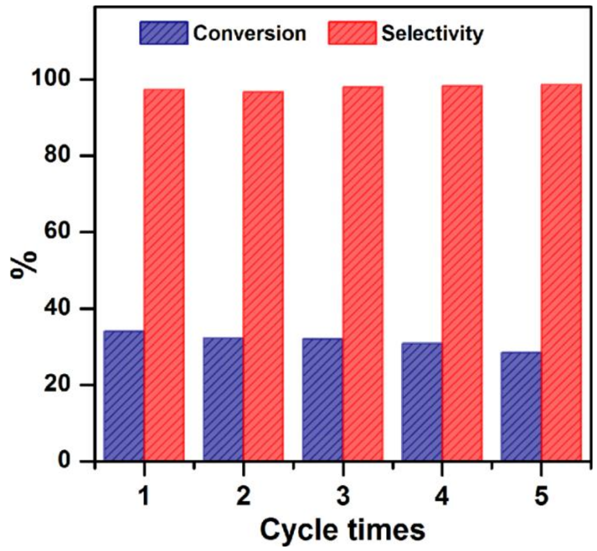


**Supplementary Figure 17**. The conversion of 3-nitrostyrene and the selectivity to 3-vinylaniline in different cycles.

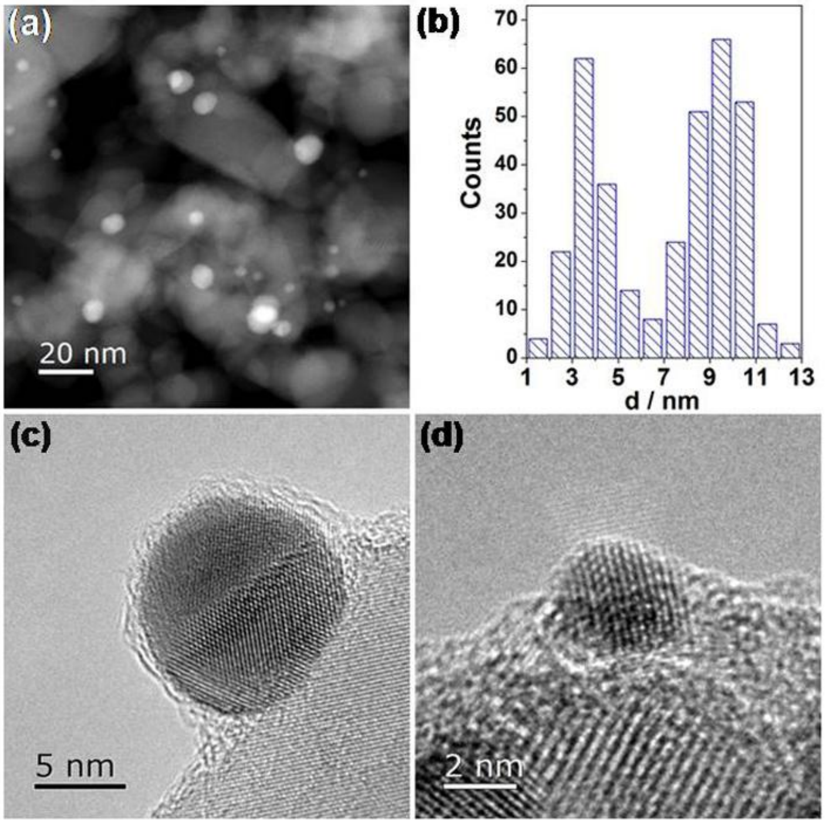


**Supplementary Figure 18**. The (a) HAADF-STEM image and (b) corresponding Au particle size distribution of Au-3+9nm-H400 sample after hydrogenation reation; and the representative HRTEM image of the (c) large and (d) small particles in Au-3+9nm-H400 after hydrogenation reaction.

**Supplementary Table 3**. Chemoselective hydrogenation of 3-nitrostyrene using different catalysts

| Entry | Catalyst | Reaction time (h) | Conv. (%) | Sel. (%) [d] | TOF ($mol_{conv.}$ $h^{-1}$ $mol_{Au}^{-1}$) |
|---|---|---|---|---|---|
| 1 | Au-3nm-H400 [a] | 1.5 | 5.2 | 92.5 | 7.1 |
| 2 | Au-9nm-H400 [b] | 4 | 3.5 | 100 | 1.3 |
| 3 | Au-3+9nm-H400 [c] | 0.5 | 4.2 | 96.3 | 11.9 |

Reaction condotions: $T$ =110 °C, $P_{H2}$ = 1.0 MPa; 3 ml reaction mixture: 0.39 mmol of 3-nitrostyrene, toluene as solvent, o-xylene as internal standard.

[a] 40 mg of catalyst, with Au loading of 0.51%.

[b] 20 mg of catalyst, with Au loading of 1.45%.

[c] 20 mg of catalyst, with total Au loading of 1.46%.

[d] the selectivity for 3-vinylaniline.

**Supplementary Table 4**. Chemoselective hydrogenation of 3-nitrostyrene using pure $TiO_2$ (P25)

| Entry | Catalyst | Conv. (%) [c] | Selectivity (%) [c] | | |
|---|---|---|---|---|---|
| | | | A | B | C |
| 1 | $TiO_2$ (P25) [a] | 0 | 0 | 0 | 0 |
| 2 | $TiO_2$ (P25)-H400 [b] | 0 | 0 | 0 | 0 |

A, B, and C represent 3-vinylaniline, 3-ethylnitrobenzene, and 3-ethylaniline, respectively. [a] 30 mg of pure $TiO_2$ (P25), reacted at 110 °C for 4h; [b] 30 mg of $TiO_2$ (P25)-H400 reacted at 110 °C for 4h. The $TiO_2$ (P25)-H400 sample was obtained by reducing $TiO_2$ (P25) under 10 vol% $H_2$/He for 1 h with a flow rate of 33.3 mL/min at 400 °C. [c] Determined by GC.

## Supplementary References


1 Rogers, C. *et al.* Synergistic enhancement of electrocatalytic $CO_2$ reduction with gold nanoparticles embedded in functional graphene nanoribbon composite electrodes. *J. Am. Chem. Soc.* **139**, 4052-4061, (2017).

2 Li, D. *et al.* Surfactant removal for colloidal nanoparticles from solution synthesis: the effect on catalytic performance. *ACS. Catal.* **2**, 1358-1362, (2012).

3 Riyajan, S.-A. & Sasithornsonti, Y. Chemical crosslink degradable PVA aqueous solution by potassium persulphate. *J. Polym. Environ.* **21**, 472-478, (2013).

4 Tang, Y., Pang, L. & Wang, D. Preparation and characterization of borate bioactive glass cross-linked PVA hydrogel. *J. Non-Cryst. Solids* **476**, 25-29, (2017).

5 Chiesa, M., Paganini, M. C., Livraghi, S. & Giamello, E. Charge trapping in TiO2 polymorphs as seen by Electron Paramagnetic Resonance spectroscopy. *Phys. Chem. Chem. Phys.* **15**, 9435-9447, (2013).

6 Hurum, D. C., Agrios, A. G., Gray, K. A., Rajh, T. & Thurnauer, M. C. Explaining the enhanced photocatalytic activity of Degussa P25 mixed-phase $TiO_2$ using EPR. *J. Phys. Chem. B* **107**, 4545-4549, (2003).

7 Nakamura, I. *et al.* Role of oxygen vacancy in the plasma-treated $TiO_2$ photocatalyst with visible light activity for NO removal. *J. Mol. Catal. A-Chem.* **161**, 205-212 (2000).

8 Okumura, M., Coronado, J. M., Soria, J., Haruta, M. & Conesa, J. C. EPR Study of CO and $O_2$ Interaction with Supported Au Catalysts. *J. Catal.* **203**, 168-174, (2001).

9 Conesa, J. C., Malet, P., Munuera, G., Sanz, J. & Soria, J. Magnetic resonance studies of hydrogen-reduced rhodium/titanium dioxide catalysts. *J. Phys. Chem.* **88**, 2986-2992, (1984).

10 Livraghi, S., Chiesa, M., Paganini, M. C. & Giamello, E. On the nature of reduced states in titanium sioxide as monitored by electron paramagnetic resonance. I: the Anatase case. *J. Phys. Chem. C* **115**, 25413-25421, (2011).

11 Livraghi, S. *et al.* Nature of reduced sates in titanium dioxide as monitored by electron paramagnetic resonance. II: rutile and brookite cases. *J. Phys. Chem. C* **118**, 22141-22148, (2014).

12 Henry, C. R. Morphology of supported nanoparticles. *Prog. Surf. Sci.* **80**, 92-116, (2005).

13 Wei, X. *et al.* Geometrical structure of the gold-iron(III) oxide interfacial perimeter for CO oxidation. *Angew. Chem. Int. Ed.* **57**, 11289-11293, (2018).

14 Jiang, Q. & Lu, H. M. Size dependent interface energy and its applications. *Surf. Sci. Rep.* **63**, 427-464 (2008).

15 Jiang, Q., Lu, H. M. & Zhao, M. Modelling of surface energies of elemental crystals. *J. Phys.- Condens. Mat.* **16**, 521 (2004).

16 Lu, H. M. & Jiang, Q. Size-dependent surface energies of nanocrystals. *J. Phys. Chem. B* **108**, 5617-5619 (2004).

17 Tyson, W. R. & Miller, W. A. Surface free energies of solid metals: Estimation from liquid surface tension measurements. *Surf. Sci.* **62**, 267-276 (1977).

18 Aqra, F. & Ayyad, A. Surface energies of metals in both liquid and solid states. *Appl. Surf. Sci.* **257**, 6372-6379 (2011).

19 Ranade, M. R. et al. Energetics of nanocrystalline $TiO_2$. *P. Natl. Acad. Sci. USA* **99 Suppl 2**, 6476-6481 (2002).

20 Zhang, H. & Banfield, J. F. Understanding polymorphic phase transformation behavior during growth of nanocrystalline aggregates: Insights from $TiO_2$. *J. Phys. Chem. B* **104**, 3481-3487 (2000).

21 Terwllliger, C. D. & Chiang, Y.-M. Measurements of excess enthalpy in ultrafine-grained titanium dioxide. *J. Am. Ceram. Soc.* **78**, 2045-2055 (1995).

22 Overbury, S. H., Bertrand, P. A. & Somorjai, G. A. Surface composition of binary systems. Prediction of surface phase diagrams of solid solutions. *Chem. Rev.* **75**, 547-560 (1975).

23 Tolman, R. C. The effect of droplet size on surface tension. *J. Chem. Phys.* **17**, 333-337, (1949).

24 Xiong, S. et al. Modeling size effects on the surface free energy of metallic nanoparticles and nanocavities. *Phys. Chem. Chem. Phys*. **13**, 10648-10651 (2011).

25 Guenther, G. & Guillon, O. Models of size-dependent nanoparticle melting tested on gold. *J. Mater. Sci.* **49**, 7915-7932 (2014).

26 Samsonov, V. M., Chernyshova, A. A. & Sdobnyakov, N. Y. Size dependence of the surface energy and surface tension of metal nanoparticles. *Bull. Russ. Acad. Sci: Phys.* **80**, 698-701, (2016).

27 Ali, S., Myasnichenko, V. S. & Neyts, E. C. Size-dependent strain and surface energies of gold nanoclusters. *Phys. Chem. Chem. Phys.* **18**, 792-800, (2016).

28 Liu, J., Papadakis, R. & Li, H. Experimental observation of size-dependent behavior in surface energy of gold nanoparticles through atomic force microscope. *Appl. Phys. Lett.* **113**, 083108, (2018).

29 Molleman, B. & Hiemstra, T. Size and shape dependency of the surface energy of metallic nanoparticles: unifying the atomic and thermodynamic approaches. *Phys. Chem. Chem. Phys.* **20**, 20575-20587, (2018).

30 Vollath, D., Fischer, F. D. & Holec, D. Surface energy of nanoparticles - influence of particle size and structure. *Beilstein J. Nanotechnol.* **9**, 2265-2276, (2018).

31 Shuttleworth, R. The surface tension of solids. *Proc. Phys. Soc. A* **63**, 444-457, (1950).

32 Jiang, Q., Zhao, D. S. & Zhao, M. Size-dependent interface energy and related interface stress. *Acta Mater.* **49**, 3143-3147, (2001).

33 Zhang, S. *et al.* Dynamical observation and detailed description of catalysts under strong metal-support interaction. *Nano Lett.* **16**, 4528-4534, (2016).

34 Tauster, S. J., Fung, S. C. & Garten, R. L. Strong metal-support interactions. Group 8 noble metals supported on titanium dioxide. *J. Am. Chem. Soc.* **100**, 170-175, (1978).

35 Zhang, L., Zhou, M., Wang, A. & Zhang, T. Selective hydrogenation over supported metal catalysts: from nanoparticles to single atoms. *Chem. Rev.* **120**, 683-733, (2020).
